# Debug Your Bugs – How NLRs Shape Intestinal Host-Microbe Interactions

**DOI:** 10.3389/fimmu.2013.00479

**Published:** 2013-12-27

**Authors:** Simone Lipinski, Philip Rosenstiel

**Affiliations:** ^1^Institute of Clinical Molecular Biology, Christian-Albrechts-University of Kiel, Kiel, Germany

**Keywords:** NLR, Crohn disease, intestinal mucosa, mucosal immunity, gut microbiota, inflammation, symbiosis

## Abstract

The host’s ability to discriminate friend and foe and to establish a precise homeostasis with its associated microbiota is crucial for its survival and fitness. Among the mediators of intestinal host-microbe interactions, NOD-like receptor (NLR) proteins take center stage. They are present in the epithelial lining and innate immune cells that constantly monitor microbial activities at the intestinal barrier. Dysfunctional NLRs predispose to intestinal inflammation as well as sensitization to extra-intestinal immune-mediated diseases and are linked to the alteration of microbial communities. Here, we review advances in our understanding of their reciprocal relationship in the regulation of intestinal homeostasis and implications for intestinal health.

## Introduction

The intestinal epithelium is the largest barrier organ of the human body and the colon harbors the majority of the individual’s microbiota (1). It is estimated that more than 1000 different bacterial species colonize the human gut, outnumbering eukaryotic cells at least by an order of magnitude (2). As many of the bacteria represent facultative pathogens (pathobionts), the integrity of the intestinal barrier must be highly secured. This is accomplished by physical and immunological mechanisms formed by cellular (i.e., epithelial- and mesoderm-derived immune cells) and non-cellular components (e.g., antimicrobial peptides, cytokines, and mucus). On the other hand, an extensive crosstalk between host and microbiota contributes to the normal development and maturation of the intestinal epithelium and immune system (3, 4). The recognition of this complex bacterial community is mediated by phylogenetically ancient innate immune receptors, e.g., Toll-like receptors (TLRs) and NOD-like receptors (NLRs). NLR proteins have co-evolved with intestinal microbial communities and are expressed by intestinal epithelial and immune cells. They are characterized by a central nucleotide-binding and oligomerization domain (NOD or NACHT) and C-terminal leucine-rich repeats (LRRs) (5). Upon activation, NLRs initiate assembly of the inflammasome or signaling cascades [e.g., NF-κB, reactive oxygen species (ROS)] leading to a transient pro-inflammatory environment and, ultimately, aim at resolution of inflammation. Dysfunctional NLR signaling is linked to intestinal inflammation and in fact, polymorphisms in NLR genes are associated with complex chronic inflammatory barrier diseases, such as inflammatory bowel disease (IBD) (6). The two major forms of IBD, Crohn’s disease (CD) and ulcerative colitis (UC) are chronic relapsing-remittent or progressive inflammatory conditions that affect the gastrointestinal tract.

It has become clear that NLRs play a crucial role for the maintenance of structural and functional composition of the intestinal microbiota. Several lines of evidence have been presented that link dysfunctional NLR signaling to an impaired host-microbiota homeostasis that may predispose to subsequent altered inflammatory responses in animal models. Here, we summarize multiple levels of host-microbe crosstalk in the intestine and review the recent findings and consequences of NLRs in physiological and pathological intestinal host-microbe interactions.

## The Role of NLRs in the Multiple Levels of Intestinal Host-Microbe Crosstalk – The NOD2 Example

The importance of NOD2 for intestinal homeostasis is emphasized by the finding that genetic variants in NOD2 contribute to dysregulated intestinal inflammatory responses and to manifestation of CD in humans. The three most common single nucleotide polymorphisms (SNPs) are located within the LRR of NOD2 causing either a frameshift mutation (L1007fsinsC), which leads to a truncated LRR or amino acid changes (R702W and G908R) (7–9). Cells that express these variants fail to activate NF-κB upon stimulation with the NOD2 ligand muramyl-dipeptide (MDP) (10, 11). In mouse models of intestinal inflammation, NOD2 has been assigned a protective role, since lack of NOD2 conferred increased susceptibility to DSS and TNBS-induced colitis (12). It must be emphasized that the effects are modest and under regular animal housing conditions no spontaneous inflammatory phenotype has been observed. Although it is still unclear how exactly a loss of NOD2 function predisposes to CD, several mechanisms related to altered host-microbe interactions and consequently increased susceptibility to intestinal inflammation, are currently discussed.

### Tolerance, polarity, and control of protective cellular programs in IECs

**Figure 1:**
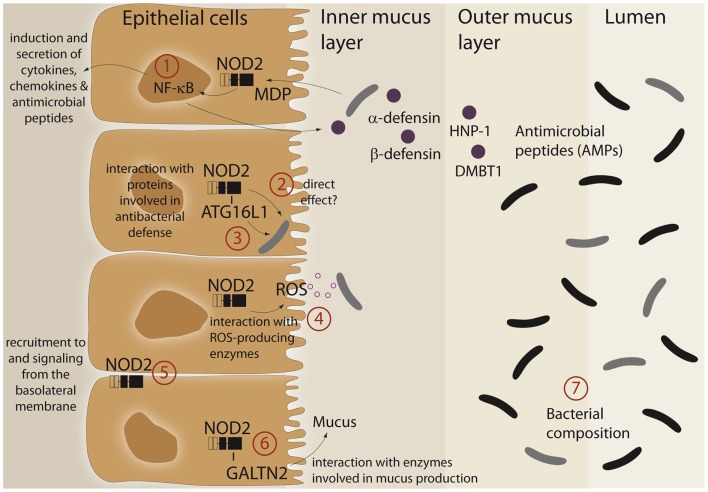
**Schematic representation of different functional aspects of the NLR family member NOD2 at the epithelial barrier**. (1) Recognition of MDP leads to a complex protective gene expression program including the induction of antimicrobial peptides and cytokines. (2, 3) NOD2 has been shown to interact with the autophagic pathway and may direct xenophagy in a direct manner. A direct antibacterial effect of NOD2 itself has been postulated. (4) There is interaction between NOD2 activation and ROS-generating enzyme complexes that may have an effect on intestinal bacteria. (5) A complex machinery regulates the presence of NOD2 at the plasma membrane (e.g., Erbin and FRMPD2) which may modulate the ability to recognize and act against invasive bacteria. (6) A recent study has shown that NOD2 is involved in mucus generation via modulation of GALNT2. For further details see main text. (7) All aforementioned factors may causally contribute to the reported differences in microbiome composition. For further details see text.

An imprinting function of NOD2 on microbial composition and/or active antibacterial responses against pathogens may be explained by its ability to modulate cellular programs in IECs (summarized in Figure 1). Furthermore, it was demonstrated that the LRR domain of NOD2 already confers antibacterial properties *per se*. The purified NOD2 LRR domain directly interacted with bacteria leading to bacterial killing, whereas the LRR domains bearing the CD-associated mutation L1007fsinsC lacked antibacterial activity (13). Moreover, NOD2 exhibits additional antibacterial effects by interacting with various proteins, which have been implicated in bacterial clearance. Of these, ATG16L1, a protein involved in antibacterial autophagy (“xenophagy”), was shown to interact with NOD2 and to cooperatively mediate pathogen defense in intestinal epithelial cells (14–16). This is of interest since variants in ATG16L1 are associated with CD (17) and combination of disease-associated alleles of ATG16L1 and NOD2 are assumed to synergistically increase susceptibility for CD (18, 19). Moreover, NOD2 was shown to interact with both components and catalytic proteins of ROS-producing enzymes. ROS production is an integral part of the innate host defense system, and inflammatory responses at mucosal surfaces include moderate (activation of signaling cascades) to excessive (bacterial killing due to oxidative burst) formation of ROS. Intestinal epithelial cells express members of the ROS-generating NADPH-oxidase complex (20) and MDP induces ROS formation (21, 22). NOD2 was shown to interact with the structural NADPH-oxidase component Rac1 (23, 24) and with the DUOX family member DUOX2 (22). Another important facet in the regulation of NOD2 signaling is the specific localization within the intestinal epithelial cell. Despite its intracellular localization, NOD2 can shuttle to the basolateral plasma membrane upon activation (25–27). Moreover, NOD2-mediated cytokine release and defensin production are specifically induced from a membrane complex including Erbin and FRMPD2 from the basolateral side (28).

A link between NOD2 and intestinal mucus production has been established with the discovery that NOD2 interacts with GALNT2 (polypeptide *N*-acetylgalactosaminyltransferase 2), a regulator of mucin biosynthesis. A defect in GALNT2 function due to impaired NOD2 interaction might therefore alter mucin production and hence contribute to CD susceptibility (29).

NOD2 signaling leads to activation of NF-κB and subsequent induction of diverse antimicrobial peptides and proteins like HNP-1 (30), β-defensin-2 (28, 31), and DMBT1 (32), a Scavenger Receptor Cysteine-Rich (SRCR) domain-containing protein, which interacts with and agglutinates several Gram-negative and Gram-positive bacteria [reviewed in Ref. (33)]. Since patients with ileal CD exhibit reduced levels of Paneth cell derived α-defensins HD-5 and -6 (34, 35) and NOD2 is constitutively expressed by Paneth cells (36), several studies investigated an underlying causal role for NOD2. However, contradictory results exist. Whereas patients carrying NOD2 polymorphism had greater reduction of α-defensins (37, 38), no genotype-dependent correlation was found in another study (39). Similarly, conflicting data exists from transgenic mouse models. Nod2-deficient mice displayed reduced mRNA expression of α-defensins compared to wild-type mice (40). These results were challenged by the recent finding that NOD2 knockout mice that were co-housed with their wild-type littermate had equivalent α-defensin profiles and identical antimicrobial activity against commensal and pathogenic bacterial strains (41). Moreover, NOD2-deficient mice were not impaired in Paneth cell numbers compared to wild-type animals (42). Thus, further work needs to clarify the role of NOD2 in regulating mouse α-defensin status (42).

### Effects on microbial composition

**Figure 2:**
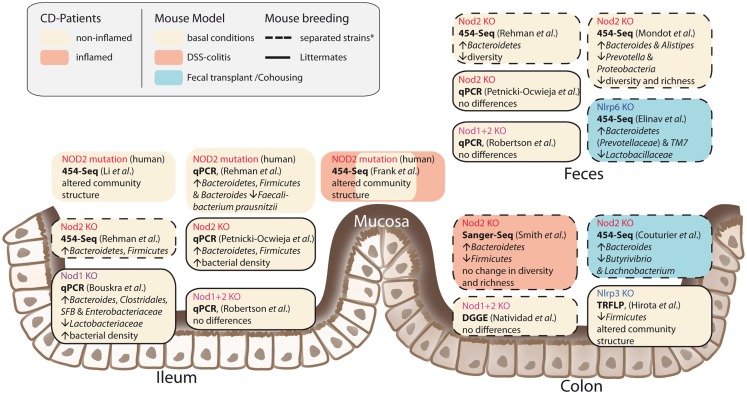
**Influences of NLRs on intestinal microbial community structures**. The figure summarizes recent studies in humans and mice and depicts different approaches and animal breeding schemes. The asterisk denotes the fact that behind the term separated breeding a variety of strategies is conjoined. For further discussion see main text.

It has been shown that NOD2 is involved in recognition and defense against various intestinal pathogens, including *Helicobacter pylori* (43), *Helicobacter hepaticus* (44), *Citrobacter* (45), *Salmonella typhimurium* (46), *E. coli* (47), and *Listeria monocytogenes* (40, 48). In CD-affected humans, the link between NOD2 status and intestinal dysbiosis has been confirmed in disease patients homozygous for the NOD2 L1007fsinsC mutation. Tissue-attached microbiota from ileal biopsies exhibited higher loads of *Bacteroidetes*, *Firmicutes*, and *Bacteroides* compared to healthy controls. In fecal samples, a similar pattern was observed, however differences were not statistically significant (49). Another study that incorporated CD patients mutated in one of the three major risk alleles (R702W, G908R, and L1007fsinsC) confirmed that genotype and disease phenotype are associated with shifts in their intestinal microbial compositions (50). Nevertheless, NOD2-deficient mice do not develop spontaneous colitis when kept under specific pathogen free (SPF) conditions. With the advent of next-generation sequencing, it has become possible to take an in-depth snapshot of the intestinal bacterial ecosystem and to delineate microbial community structures and composition at the species level. However, considerable differences between published studies exist concerning animal housing and breeding (e.g., hygiene status of animal facility, genetic background, caging effects, use of F2 littermates, or separated WT/knockout strains) study design (age, sex, intestinal sampling location) and sequencing methods (DNA extraction, sequencing, and data analysis). Despite these differences, several independent groups reported that NOD2 status is associated with alterations in the intestinal microbial composition and density (summarized in Figure 2) (47, 49, 51, 52). Increased abundance of members of the phylum *Bacteroidetes* was detected in weaning mice and persisted throughout development (49). In line with this, RIPK2-deficient mice displayed increased levels of *Bacteroides* and *Firmicutes* arguing for a RIK2-dependency (47). Greater fecal abundances within the *Alistipes* and *Bacteroides* but an underrepresentation of *Prevotellacea* along with a decreased diversity and richness in the microbiota was found in NOD2^−/−^ compared to WT mice (51). Recently, another aspect of the complex host genotype-microbe interaction was highlighted. Wild-type mice that received disease-predisposing bacterial communities from NOD2 or RIPK2-deficient mice via co-housing or cross-fostering experiments suffered from increased susceptibility to DSS-induced colitis and colitis-associated carcinogenesis. Reciprocal microbiota transplantation from wild-type donors reduced disease risk in NOD2-deficient mice (53). However, two recent studies reported only minimal differences in gut microbial composition of co-housed, littermate controlled NOD2-deficient, and wild-type mice (41, 54). The latter one showed that shifts in bacterial communities were independent of genotype and correlated with housing conditions (54). In light of the findings from recent co-housing experiments with NOD2 and other NLRs [e.g., Ref. (53, 55)] this might be partly explained by the restoration of disturbed microbiota due to animal co-housing, however, more studies are needed to fully understand the interference of NOD2 with host-microbe interactions.

## NOD all NODs are Created Equal – Lessons from NOD1

NOD1 and 2 share similar structural composition, detection of peptidoglycan moieties (iE-DAP/NOD1, MDP/NOD2), and downstream signaling pathways, including RIPK2 and NF-κB activation. In contrast to NOD2, the association between genetic variants in the *NOD1* gene and susceptibility to IBD is less evident. While some studies identified *NOD1* as a risk factor for IBD in some studies (56, 57) this has not been widely replicated (58–60) including a recent meta-analysis (61).

Nevertheless, there is evidence that NOD1-mediated innate immune responses are critically involved in maintaining intestinal homeostasis. Depletion of intestinal microbiota was associated with impaired neutrophil function, which was reversed by administration of NOD1 ligand in the drinking water of mice (62). Moreover, NOD1-deficiency leads to increased susceptibility to *H. pylori* infection (63), impaired clearance of *Clostridium difficile* in the intestine, increased bacterial translocation (64), and enhanced colitis-associated colon tumor formation (65). The NOD1-mediated recognition of peptidoglycan was necessary to induce genesis of isolated lymphoid follicles (ILFs) in the intestine, which in turn influenced the composition of the intestinal bacterial community. In NOD1-deficient mice, the total bacterial population was expanded 100-fold, which was largely due to the groups of *Clostridiales*, *Bacteroides*, and *Enterobacteriaceae* (66). Furthermore, lack of NOD1 led to deficiencies in intestinal barrier integrity reflected by lower expression levels of NOD2, Muc2, α- and β-defensin, and keratinocyte-derived chemokine (KC) as compared to their F2 littermates (54). In line with this, the combined knockout of NOD1 and 2 led to increased paracellular permeability, decreased levels of E-cadherin, and lower colonic antimicrobial RegIII-γ expression in comparison to littermate control mice (67). Nevertheless, both studies did not find genotype-specific differences in the relative abundance of intestinal bacteria (54, 67).Thus, as previously pointed out for NOD2, the impact of breeding strategies and housing conditions may strongly interfere with study results and yet it is still too early to draw final conclusions about the role of NOD1 in physiological and pathological host-microbe interactions in the intestine.

## IL-1β – the Role of Inflammasome-Type NLRs in the Intestine

Several NLRs form multimeric complexes termed “inflammasomes” that serve as molecular platforms for caspase-1 activation and processing of pro-IL-1-like cytokines into their active forms (68). Until now, this group comprises the NLRPs (NLRs with PYRIN domain) NLRP3, NLRP6, NLRP1, NLRP12, NLRP7, and NLRC4 (69, 70). Although no variants in inflammasome forming NLR genes are among the 163 IBD susceptibility loci (71), their relevance for intestinal health has been shown by various inflammasome-deficient mice in models of intestinal inflammation, as reviewed in Ref. (72). However, in comparison to mice deficient for Nlrp3, Nlrp10, Nlrp12, and Nlrc4, Nlrp6 showed the largest potential to alter microbiota and colitis susceptibility of co-housed mice. In the following paragraph we will therefore focus on the role of Nlrp6 in intestinal host-microbe interactions.

Components of the Nlrp6 inflammasome are expressed in intestinal epithelial cells (73) and throughout the intestinal tract (55), and several studies have demonstrated a protective role of Nlrp6 against colitis and colitis-associated tumor formation (55, 73–75) [reviewed in Ref. (76)]. Importantly, *Nlrp6*-deficiency was demonstrated to significantly alter intestinal microbiota composition (55). On the genus level, *Prevotellaceae* (belonging to the *Bacteroidetes* phylum) were strongly increased, whereas *Lactobacilli* (*Firmicutes* phylum) were decreased. In addition, members of the phylum of TM7, which were highly abundant in *Nlrp6*-deficient mice, have been found to be overrepresented in CD patients (77). Likewise, *Prevotellaceae* were more prominent in the mucosa tissues of patients with UC compared to healthy individuals (78). The distinct bacterial composition of *Nlrp6*-knockout mice was transmissible to co-housed adult mice and cross-fostered litters and resulted in colitis-prone phenotype of recipient wild-type mice. Similarly, mice deficient in the inflammasome adaptor Asc harbored a colitogenic gut microbiota that was transmissible to co-housed WT mice (79). Wild-type mice exhibited increased colonic Il-6 levels compared to single-housed wild-type mice when they were co-housed with either *Nlrp6*- or *Asc*-deficient mice. Of note, the microbiota-mediated transmissible cell proliferation and tumor formation were abrogated when either a neutralizing anti-IL-6 receptor antibody was administered or intestinal IL-6 receptor was conditionally deleted in intestinal epithelial cells. Recently, the role of Nlrp6 for colonic health was extended to the small intestine (80). In a mouse model for small-bowel inflammation, stress-mediated release of corticotropin-releasing hormone (CRH) inhibited intestinal *Nlrp6* (but not *Nlrp3*) expression and altered the composition of the intestinal microbiota, which ultimately led to intestinal inflammation (80). Together, these studies point to a critical role for the NLR-forming inflammasomes, in particular Nlrp6, in modulating intestinal homeostasis via an influence on microbiota composition. It must be emphasized that anti-IL-1 treatment, despite having an effect in DSS colitis, lacks efficacy in IBD. With the above knowledge in mind, this field is now at a point where the translation into the human situation is desperately needed.

## Conclusion

We are beginning to realize that maintaining the long-term stability of the co-evolved human gut microbe communities is an important mechanism for maintaining human health. The ecology of intestinal microbiota is not only necessary for digestion of nutrients and the delivery of local metabolites (e.g., butyrate) to intestinal epithelial cells but also critically shapes immune responses of the host. An important fact for future studies will be to delineate how this interaction licenses migratory immune cells for functions in other organ systems such as the brain. It must be emphasized that most of our knowledge of the role of NLRs for this symbiotic relationship originates from animal studies in rodents and that there are also conflicting results in terms of the extent of the influence of single NLRs in this context. Beyond the biology of NLRs, these results teach us two things: (i) we have to reassess how we set up our immunological animal models in the future. From findings that susceptibility to provocation models may be transmissible by genetically determined microbiota to wild-type animals, it is clear that a regular F2 intercross with littermate housing may not be an ideal scenario. On the other hand, drift in microbiota in separated lines over generations may exert bigger effects than the actual genotype. This is a dilemma we have to solve. (ii) We have to be careful how we interpret the findings. In the end only the transfer into the human situation will help us to understand the factual influence of microbiota on traits in health and disease.

Taken together, NLRs represent watchful guardians at the intestinal barrier, which help to maintain immunological homeostasis in an organ system facing strong environmental influences. This environment has changed drastically over the past 100 years and some NLR family members are clearly involved in chronic inflammatory diseases associated with this lifestyle. Decoding the exact molecular signals of NLRs that contribute to the resilience of microbial communities on mucosal surfaces may provide approaches to prevent or ameliorate this range of human diseases. More than 10 years into NLR research we are still far away from understanding how these molecules actually exert their function and how we can target them in therapy.

## Conflict of Interest Statement

The authors declare that the research was conducted in the absence of any commercial or financial relationships that could be construed as a potential conflict of interest.
